# Weather induced post failure kinematics of a highway embankment founded on a marly sandstone slope

**DOI:** 10.1038/s41598-023-49181-3

**Published:** 2023-12-09

**Authors:** Luca Comegna, Alessandro Mandolini, Denise Manna, Guido Rianna, Alfredo Reder

**Affiliations:** 1 Dipartimento di Ingegneria, Università degli Studi della Campania “Luigi Vanvitelli”, Via Roma 29, 81031 Aversa, Italy; 2 Fondazione Centro Euro-Mediterraneo sui Cambiamenti Climatici, Regional Models and Geo-Hydrological Impacts (REMHI) Division, Via Thomas Alva Edison, 81100 Caserta, Italy

**Keywords:** Civil engineering, Natural hazards

## Abstract

A highway embankment founded on a sloping tectonised marly-sandstone flysch formation located in the Apennines chain (Italy) has been affected for about 30 years by continuous slow movements. Given the strategic importance of the involved infrastructure, different investigation and monitoring campaigns have been carried out to get information about the properties of the involved soils and collect data about the displacements and piezometric regime. Field monitoring, in particular, reveals that the observed displacements result from a failure mechanism involving both the embankment and the foundation soils. However, significant gaps in monitoring jeopardize the possibility to assess the long-term trends in the displacements and piezometric regime and the significance of weather forcing in regulating the phenomena. To address such research questions, a procedure, easily transferable in different contexts, is proposed and applied to the test case: a simple hydrological proxy indeed permits evaluating the rate of movement featured by weatherinduced seasonal variability. Such a mechanical response has been confirmed by the results of a simplified numerical model aimed at finding out the main features of the observed kinematics accounting for a hydrological balance of the involved area.

## Introduction

Many sloping areas of the Apennines chain (Italy) are frequently affected by movements involving the widely outcropping structurally complex flysch formations^1–6^. The instability of such units, consisting of fractured rocks, rock blocks or fragments surrounded by a clayey matrix, is favoured by high internal deviatoric strain induced by the intense tectonic events that led to the development of a number of often persistent discontinuities along which the available shear strength is equal to the residual value^7^. Being the involved slopes rather gently inclined, the rate of such movements is usually low, and human life is consequently not threatened. Nevertheless, experience shows that the corresponding cumulative movements can damage structures, infrastructures, and lifelines, which sometimes lose their serviceability thus inducing land managers to make decisions to reduce the associated economic losses. Referring to the specific Italian context, different case studies dedicated to the interaction between such slope movements and man-made works (such as facilities, artefacts, railway and/or highway tunnels) are reported in the literature^8–17^.

**Figure 1:**
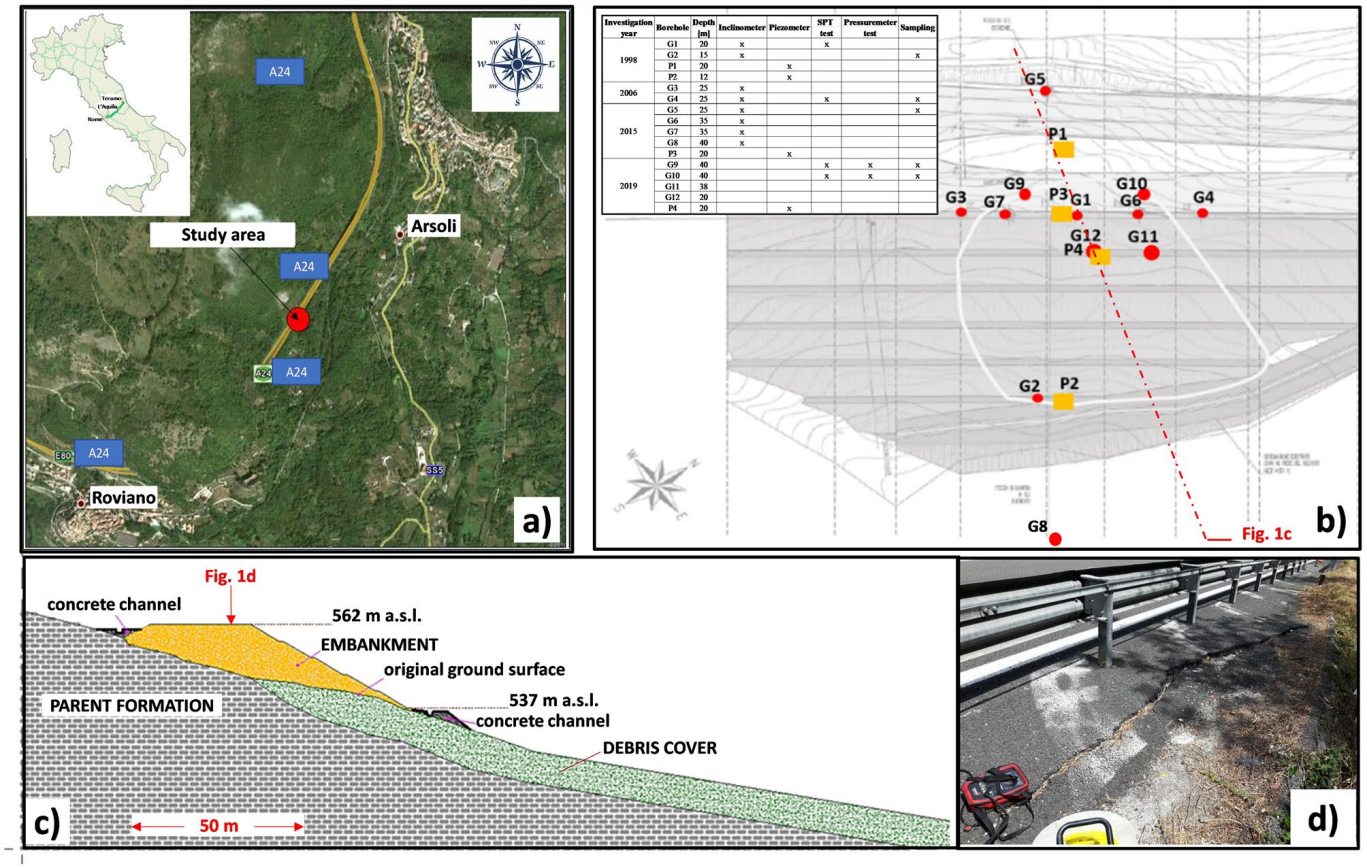
Roviano case study: (**a**) aerial photo of the site (provided by the web site https://earth.google.com); (**b**) location of the boreholes (modified after Manna^18^); (**c**) reference main section; (**d**) fracture observed on the road paving.

The object of the present paper is a road embankment section of the Italian highway A24 (Fig. 1a), crossing the Apennines in the southwest-northeast direction to connect the cities of Rome, L’Aquila and Teramo (Central Italy). The road embankment section consists of a 70 m long stretch realised on a sloping tectonised marly-sandstone flysch formation (Fig. 1b,c), located between the towns of Roviano and Arsoli (Lazio Region). It has been affected for about 30 years by continuous slow subsidence, which caused the formation of some fractures along the right edge of the slow highway lane connecting Rome to L’Aquila (Fig. 1d), requiring periodic maintenance works. Furthermore, due to the relevance of the damaged infrastructure, different investigation and monitoring campaigns (Fig. 1b) have been conducted from 1998 to 2020 to collect information about the properties of the soils, the groundwater regime and the displacement field. However, given the intrinsic challenges associated with the monitoring activities, data are often available for short periods interspersed with significant gaps. It consequently jeopardizes the evaluation of long-term trends and the possibility to assess the significance of hydro-meteorological patterns on landslide activity.

In this regard, the present work reports the main results obtained by elaborating all the collected data (section “Study area”). In particular, they showed that the observed subsidence phenomena results from a failure process involving the embankment and the sloping foundation soils. Moreover, the resulting displacements, featured by an average rate of about 1 cm/year, display at a monthly scale a seasonal trend featured by accelerating stages during the wet-cold season (usually occurring from November to May) alternated to decelerating phases occurring during the essentially dry-hot period. Finally, a simple numerical model (described in section “Analysis of the failure mechanism”) has been developed to better understand the detected mechanism, accounting for a local soil-hydrological balance concerning the interested area. In particular, the results of the analyses allowed a reliable historical reconstruction of the displacement regime.

## Study area

### Geomorphological features

The embankment was realised at the end of the 1970s between the altitudes of 562 and 537 m a.s.l. (Fig. 1c) of the most inclined portion (15°–20° tilted) of the Sabini mounts (Lazio Region, Central Italy) belonging to the Central Apennines chain. In the examined area, this natural slope develops in the NW–SE direction between the peak altitude of 966 m a.s.l. and the lowest value of 317 m a.s.l., corresponding to the Aniene river valley.

**Figure 2:**
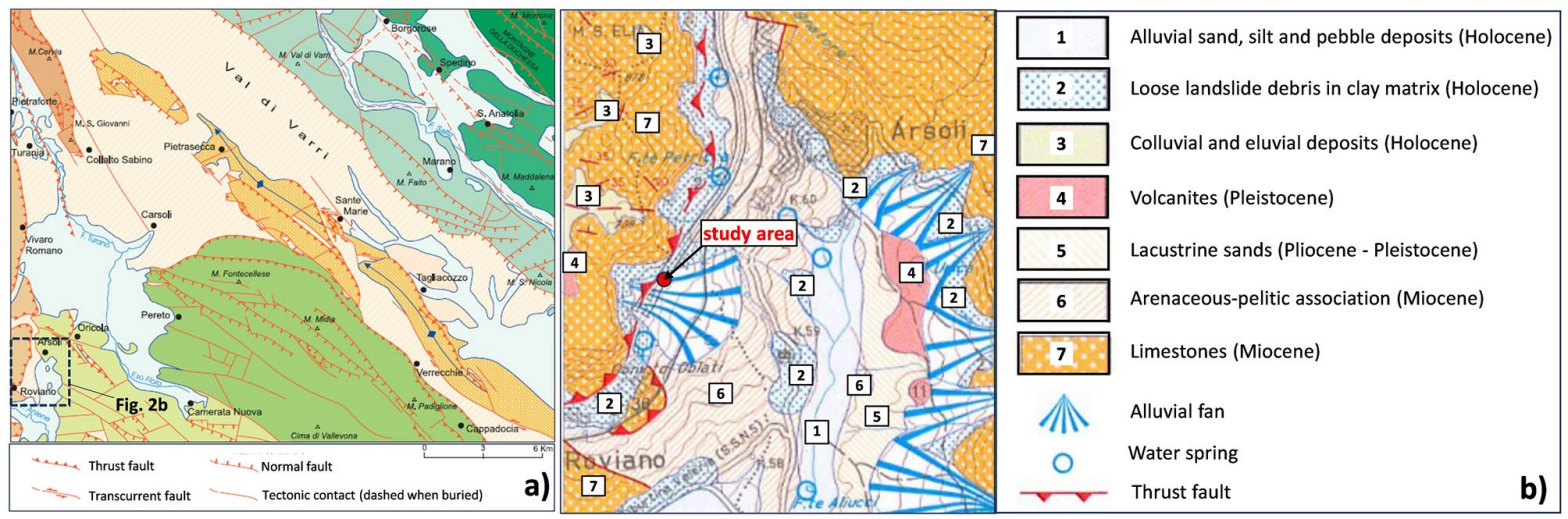
Sheet no. 367 “Tagliacozzo” of the Geological Map of Italy (provided by the website https://www.isprambiente.gov.it/Media/carg/367_TAGLIACOZZO/Foglio.html): (**a**) tectonic structural scheme; (**b**) map of the investigated area (modified after Chiarini et al.^19^).

The Central Apennines consist in a thick Triassic to Paleocene platform stratigraphic sequence followed by Early Miocene siliciclastic deposits, Late Miocene foredeep turbidites and Messinian–Early Pliocene basin deposits^19^. Concerning the tectonic activity, this chain is a NW–SE to N–S oriented thrust belt that has been continuously decomposed by both extensional and transcurrent deformations creating widespread normal and strike-slip faults (Fig. 2a), that strongly control the geomorphological evolution. Particularly regarding the examined area, as shown by the sheet no. 367 “Tagliacozzo” of the Geological Map of Italy^20^ (Fig. 2b), the involved parent formation is a Late-Middle Miocene arenaceous–pelitic association, consisting of thick to very thick, bedded and intensely laminated medium to coarse marly sandstones belonging to the “Vivaro Romano—Roviano” tectonic subunit. An upslope Early Miocene limestone thrusts over the sandstones in the same NW–SE direction of the involved slope along a reverse fault representing a continuation of the local main tectonic thrust system, known as “Olevano-Antrodoco line”^19^.

During the Late Pleistocene-Holocene age, the basal formation was partially overlain in the study area by a heterogeneous and weathered alluvial debris fan (Figs. 1c and 2b), constituted by loose silty gravels with sands and some calcareous elements.

The local geological map also reveals that the investigated area is rich in natural water springs (Fig. 2b), with discharges higher than 15 m^3^/s, as a consequence of the karst phenomena evidenced by the presence of sinkholes and caves.

Regarding the embankment, it features a maximum thickness of 16 m, average slopes inclination of 28° and an overall road width of 30 m. It is founded on both the previously described units (Fig. 1c): the upslope portion directly rests on the tectonised parent formation, while the downslope part lies on the alluvial fan.

### Soil properties

Four investigation campaigns have been conducted since 1998 to carry out information about the properties of the interested soils^21^. In particular, as shown by Fig. 1b, some boreholes were used for Standard Penetration Tests (4) and pressuremeter tests (2); five undisturbed samples were also retrieved for laboratory tests. Geological surveys and geotechnical investigations clearly revealed that three main units had been involved (Fig. 1c): the highway embankment, the basal parent formation and the alluvial debris.

**Figure 3:**
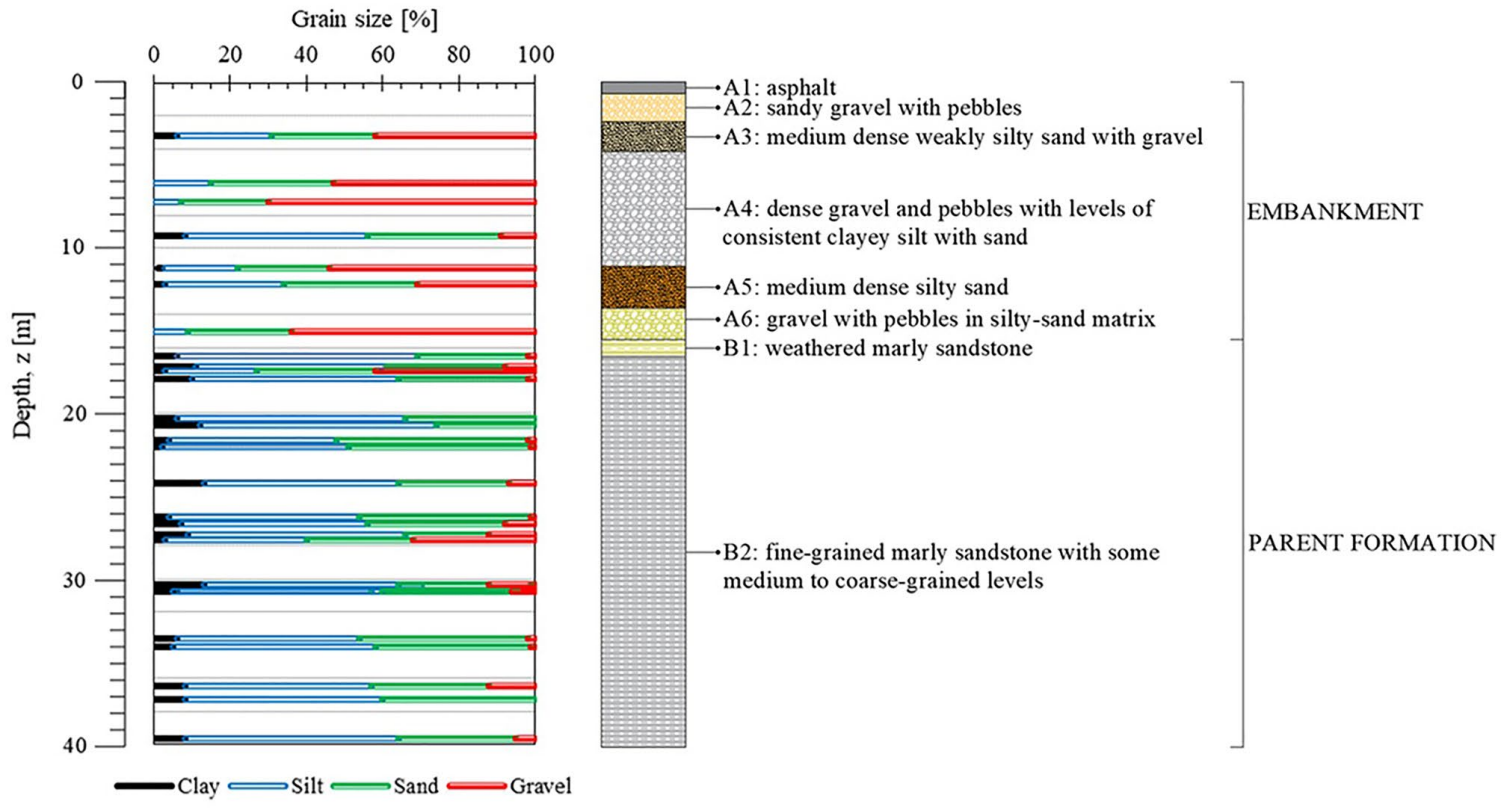
Stratigraphic column along the borehole G10 and grain size ranges.

**Figure 4:**
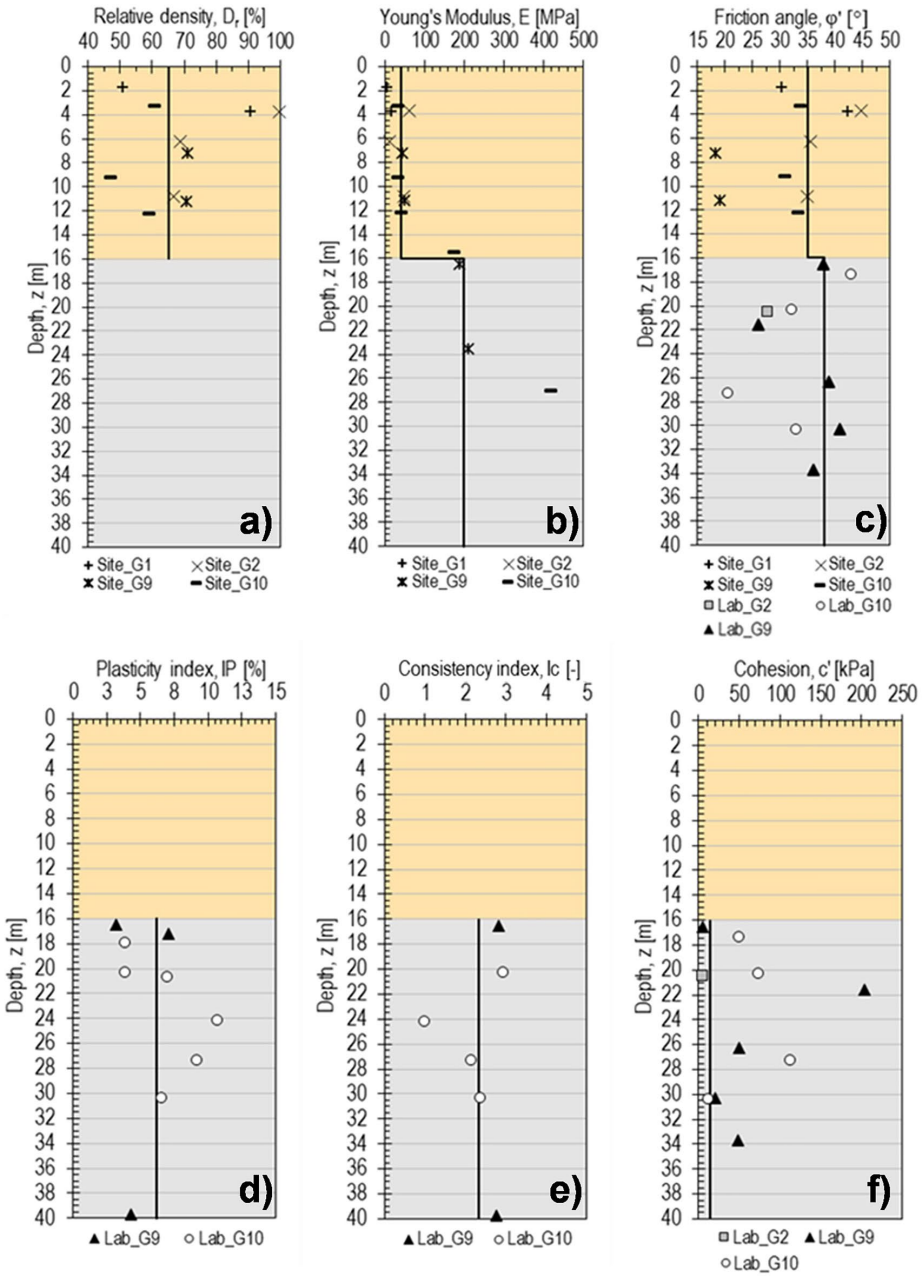
Physical and mechanical properties of the embankment (investigated depth *z* lower than 16 m) and of the parent formation (depth *z* ranging between 16 and 40 m), detected by in situ and laboratory tests: relative density, *D*_*r*_ (**a**); Young’s modulus, *E* (**b**); friction angle, *φ*′ (**c**); plasticity index, *PI* (**d**); consistency index, *Ic* (**e**); cohesion, *c*′ (**f**).

As derived by the borehole G10 (Fig. 3), the embankment is constituted by alternating medium dense to dense layers of silty-sandy gravels and silty sands. The Standard Penetration Tests carried out along the entire thickness allowed the detection of physical and mechanical properties (Fig. 4a–c). Based on some empirical correlations available in literature^22–24^, representative values for relative density (*D*_*r*_ = 0.65, Fig. 4a), Young’s modulus (*E* = 40 MPa, Fig. 4b) and friction angle (*φ*′ = 35°, Fig. 4c) have been estimated, even though scattered because of the alternation of finest layers to coarsest and densest layers.

The tectonised parent formation is a saturated clayey and gravelly silt with sand, including some blocks, as typical of flysch (Fig. 3). Some local medium to coarse grained levels are also present. The about 1 m thick shallowest part, directly in contact with the embankment, is weathered. The unit features an overall low Plasticity Index *IP* = 6% (Fig. 4d) and a very high consistency index *I*_*c*_ = 2.34 (Fig. 4e). Some oedometer tests have returned an average saturated hydraulic conductivity *k*_*sat*_ = 2.0E−10 m/s, but, as typical of structurally complex formations, a higher value of the field hydraulic conductivity (usually at least one order of magnitude) should be expected due to macrofissuring induced by tectonics^25^, whose effects, of course, can not be captured by laboratory tests on small specimens. Regarding strength parameters, a cohesion *c*′ = 14 kPa (Fig. 4f) and a friction angle *φ*′ = 37° (Fig. 4c) have been found after a linear interpolation in the Mohr–Coulomb plane of the results of 21 triaxial compression tests carried out in drained and undrained conditions on undisturbed probes (extracted from 10 samples taken at depths from 16.5 and 34 m) under an effective mean confining pressure ranging between 100 and 560 kPa. The not negligible scattering between the overall calculated *c*′ and *φ*′ values and those obtained apart from the triaxial compression tests carried out on each sample, highlighted by Fig. 4c,f, is clearly due to the nonlinear strength envelope of the investigated soil. In particular, the rather high *φ*′ value, uncommon for a fine-grained deposit, is justified by the presence of the recognised local coarser levels (Fig. 3) also including some lithoid elements, thus highlighting the complex structure of this deposit. Some direct shear tests reveal an average residual friction angle *φ*′_res_ = 20.4°, that is consistent with the low Plasticity Index of the soil^26^. Finally, a Young's modulus *E* = 200 MPa has been estimated through standard pressuremeter tests carried out at depths ranging between 16 and 27 m (Fig. 4b).

Concerning the debris cover, in situ direct observations allowed the identification of an about 8 m thick weathered gravel mixed with silty sand. However, no data about the corresponding physical or mechanical properties are available.

### Monitoring data

Information about the groundwater and displacement regime^21^ has been carried out during two different time intervals (Fig. 1b). The first monitoring stage provided data from October 1998 to July 2003, thanks to the installation of two inclinometers (G1, G2) and two open standpipe piezometers (P1, P2). The second one gave information from July 2015 to September 2020 after the installation of four inclinometers (G5, G6, G7, G8), one open standpipe piezometer (P3) and one Casagrande type piezometer (P4). Two additional inclinometers (G3 and G4) were also installed in 2006 but failed after a short period of activity. Therefore, field monitoring has been affected by a lack of data for about 11 years (from 2004 to 2014) because of unexpected technical problems and a change in the local management of the area. The main obtained results are described in the following sections.

#### Piezometric regime

**Figure 5:**
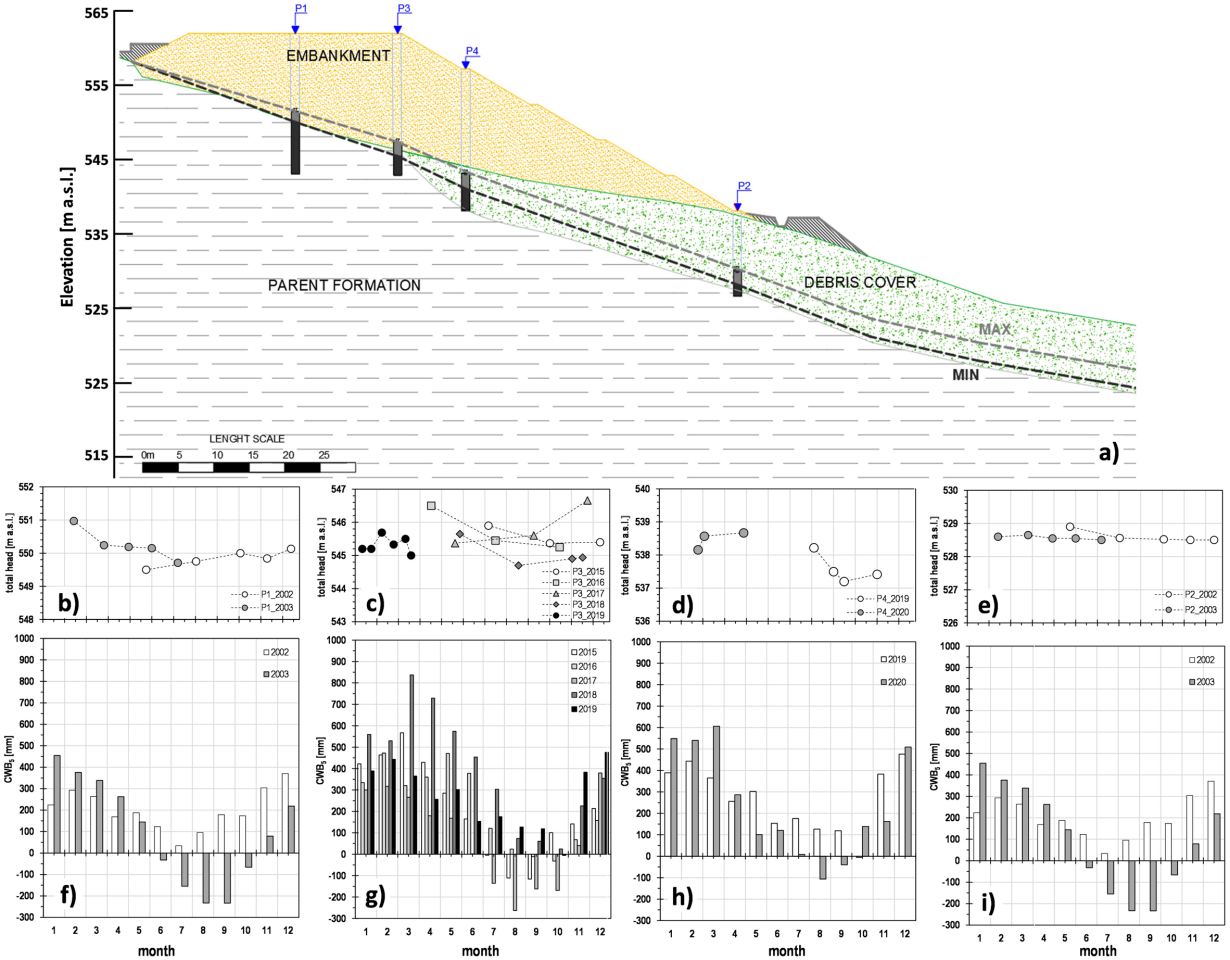
Piezometric regime: location of the installed piezometers, and monitored minimum and maximum positions of the groundwater level (**a**) (modified after Manna^18^); total hydraulic heads derived from readings of piezometers P1 from May 2002 to July 2003 (**b**), P3 from July 2015 to March 2019 (**c**), P4 from August 2019 to April 2020 (**d**) and P2 from May 2002 to July 2003 (**e**), compared to the corresponding Cumulative Water Balance calculated over 5 months, *CWB*_*5*_ (**f**–**i**).

All the four piezometers were installed within the parent formation (Fig. 5a). The three open standpipe piezometers (P1, P2 and P3) consist of 50 mm diameter PVC tubes with a 1 m long slotted section at the measurement depth. The Casagrande piezometer (P4) is featured by two 10 mm diameter PVC tubes with a 250 mm long ceramic porous filter. In all the cases, the annular space between the perforated and casing was filled with sand; seals of bentonite were inserted above and below the response zone. The two uppermost piezometers, P1 and P3, were located at a depth from the stratigraphic contact embankment/flysch of 7.5 m and 2.7 m respectively. The other two piezometers, P2 and P4, were installed at 0.5 m and 0.2 m, respectively, below the contact debris/flysch. Piezometers P1 e P2 remained operational for about 1 year (from May 2002 to July 2003). Piezometer P3 provided data over 3 years (from July 2015 to March 2019). The most recently installed piezometer P4 worked for 9 months, from August 2019 to April 2020. All the readings were manually taken by an electronic water level sounder at an average monthly frequency.

Figure 5b–e focus on details regarding the hydraulic total heads calculated as a sum of the elevation heads (referred to the mean sea level) of all the installed cells, and the pressure heads measured during the different monitoring years. According to these data, we could derive that the downslope flow within the parent formation is featured by an average gradient of about 40%, that is a little higher than the local average slope gradient (about 32%). The yearly water levels average fluctuations range from about 0.5 m (measured by piezometer P2) and around 1.5 m (measured by piezometers P3 and P4). Figure 5a shows the minimum and maximum detected positions of the groundwater level. The lowest level, usually reached during Summer from July to September (months no. 7, 8 and 9 in Fig. 5b–e), lies along the contact embankment/flysch, as derived by the uppermost piezometers P1 and P3, and about 2.0 m over the contact debris/flysch, as revealed by the lowermost P2 and P4. The highest piezometric level, usually attained during Winter from January to March (months no. 1, 2 and 3), was monitored at a distance over the parent formation, ranging between 2 m (within the embankment) and 5.1 m (within the debris cover). The phreatic surface crossing the debris is about parallel to the top of the parent formation. The high piezometric levels detected within the embankment confirm the large water availability due to the numerous water springs recognised in the study area. However, it’s worth noting that, as remarked by Picarelli et al.^27^, the presence of a network of interconnected discontinuities in structurally complex formations could also play an important role in the groundwater flow and pore water pressure regime, making difficult the corresponding interpretation.

#### Displacement regime

Displacement monitoring started in October 1998, after installing the inclinometer G1 (working for about 5 years until July 2003) in a borehole located at the right edge of the slow highway lane (Fig. 1b) connecting Rome to L’Aquila. Other information about the horizontal displacement of this side of the embankment was provided by inclinometers G6 (from February 2019 to June 2019) and G7 (from November 2017 to March 2019), whose different positions in the plan are shown in Fig. 1b. On the other hand, G5 provided data about the displacements of the left side of the embankment (during the time intervals September 2015–June 2016 and March 2019–September 2020). The remaining displacement data were given by G2 (from August 2002 to July 2003), installed at the toe of the embankment, and by G8 (from May 2018 to May 2019), located downslope at a distance of about 40 m from G2.

**Figure 6:**
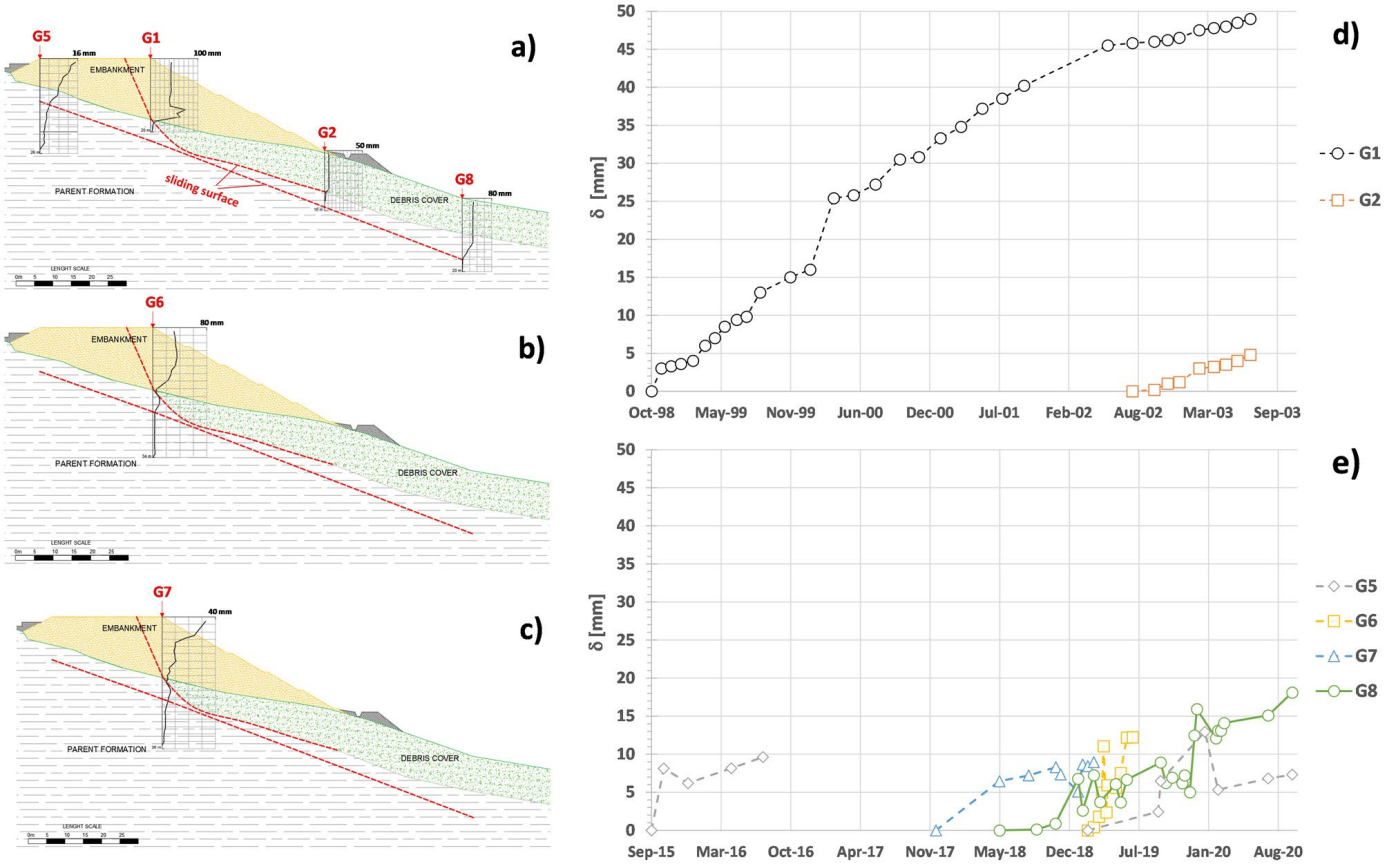
Displacement regime: detected slipping surface and location of the installed inclinometers G1, G2, G5, G8 (**a**), G6 (**b**) and G7 (**c**); horizontal displacement monitored at the ground surface by inclinometers G1 and G2 from October 1998 to September 2003 (**d**) (modified after Manna^18^), and by inclinometers G5, G6, G7 and G8 from September 2015 to September 2020 (**e**) (modified after Manna^18^).

All the monitored displacements reveal a main NW–SE direction consistent with that of the natural slope. In particular, the largest shear strains have been detected along two continuous failure surfaces at different depths (Fig. 6a–c). The shallowest slipping surface, about 80 m long, develops within the embankment and along the alluvial debris/parent formation stratigraphic contact located at the average depth of 8 m, running from the position of the fracture observed on the road paving until a distance corresponding to that of the embankment toe. The monitored deepest slipping surface consists of a 20° inclined and 120 m long plane, fully developed within the parent formation and located between 12 and 22 m. No exit points of both the slip surfaces have been observed on the ground surface. The average rates of displacement measured at the ground surface are about 10 mm/year (Fig. 6d,e).

## Evaluation of the weatherinduced impacts on slope hydrology

**Figure 7:**
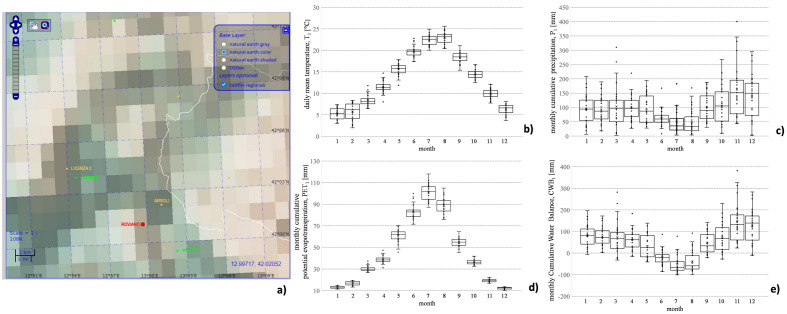
SCIA-ISPRA climate dataset: (**a**) grid mesh (provided by the website http://www.scia.isprambiente.it) and location of the four weather stations closest to the Roviano site (LAT 42°01′13″ N, LON 12°59′49″ E); collected data from 1991 to 2020 about daily mean temperature, *T*_*1*_ (**b**); monthly cumulative precipitation, *P*_*1*_ (**c**); monthly cumulative potential evapotranspiration, *PET*_*1*_ (**d**); monthly cumulative water balance, *CWB*_*1*_ (**e**).

To assess the potential role of weather variability on slope hydrology and the associated movements, characterisation is expressly focused on precipitation and temperature evolutions. Indeed, they are considered the main proxies for water inputs and losses (assuming the temperature is the primary weather forcing driving evapotranspiration phenomena) through the soil surface. Unluckily (also for weather forcing), significant temporal gaps affect observations from weather stations in the area. Specifically, the management changes from a nationally centralized to a regional one, occurred in Italy around 2000, often induced significant variations in the location of weather stations and implemented devices, consequently limiting the homogeneity of collected data. Furthermore, the average distance from the investigated slopes and the local geomorphological settings undermine the representativeness of the measures. To partly cope with such weaknesses, weather data have been retrieved by the Italian gridded land-only SCIA-ISPRA dataset, corresponding to the national system for collecting and processing climate data managed by the Italian Environmental Protection Agency. In particular, the dataset provides daily rainfall (spatial resolution = 10 km) and temperature (spatial resolution = 5 km), interpolating a large number of readings from different local weather stations^28^. Such a dataset can support long-term analysis providing spatially and temporally homogeneous and consistent information; on the other hand, the reliability and representativity of the values remain the main function of the quantity and quality of weather observations exploited to build the dataset. Concerning our case study, Fig. 7a shows the used grid mesh and the four weather stations (named Agosta, Arsoli, Licenza and Licenza 2) closest to the examined area in Roviano (LAT 42°01′13″ N, LON 12°59′49″ E). Particularly regarding the 30-year period 1991–2020, which includes available field monitoring data about piezometric regime and displacements (described in section “Monitoring data”), Fig. 7b,c show the Box–Whisker plots of daily mean temperature, *T*_*1*_ (b), and monthly cumulative values precipitation, *P*_*1*_ (c), over the time span 1991–2020. As is well known, Box–Whisker elaboration permits summarizing several information about the data distribution: the boundaries of the box represent first and third quartile, while the line in the box coincides with the median value (cross identifies the mean value). Furthermore, the whiskers extend up to the minimum (maximum) between the minimum (maximum) value of the dataseries and first quartile less (third quartile plus) 1.5⋅*IQR* (Inter Quartile Range), where *IQR* is given by the difference between third and first quartile. Furthermore, for each month, all the thirty points for the years 1991–2020 are reported.

The two variables are characterized by the typical trend retrievable in the Mediterranean area. Concerning *T*_*1*_ (Fig. 7b), the maximum values are observed in Summer season with data ranging from 20 and 25 °C in July (month no. 7) and August (month no. 8), while the minimum ones are recorded in Winter (months no. 12, 1 and 2) with values in the range between 3 and 7 °C. The monthly variability is quite limited in many cases. Regarding *P*_*1*_ (Fig. 7c), the maximum values are observed in November (month no. 11) and December (month no. 12) with a median value equal to about 150 mm; on the other hand, the 2 months are characterized by a significant interannual variability: e.g., in November, the minimum is equal to 43 mm while the maximum is 401 mm. The minimum *P*_*1*_ values are detected in July and August with a median value of about 40 mm. Moreover, Fig. 7d shows the monthly potential evapotranspiration *PET*_*1*_ calculated by converting the temperature values through the simple equation suggested by Hamon^29^:

1 $$PET_{1} = 13.97 \cdot d \cdot D^{2} \cdot W_{t}$$

where *d* is the number of days in a month, *D* is the mean monthly hours of light, and *W*_*t*_ is a saturated water vapour density term (depending in turn on mean air temperature). Given the simplified approach used to estimate *PET*_*1*_, the annual cycle reflects that for *T*_*1*_. In particular, the estimated *PET*_*1*_ values usually range from about 10 to 100 mm with values in late Autumn and Winter not exceeding 15 mm while, during the Summer, the values are always higher than 70 mm. Furthermore, interesting insights can be given by the potential soil-hydrological balance of the study area given by the difference between the monthly values of the cumulative precipitation, *P*_*1*_, and the potential evapotranspiration *PET*_*1*_

2 $$CWB_{1} = P_{1} - PET_{1}$$

where *CWB*_*1*_ is the monthly Cumulative Water Balance^30^. Precipitation and potential evapotranspiration represent the maximum potential water entering and leaving the soil. At the same time, the actual amounts depend on the soil surface hydraulic state driving infiltration and actual evapotranspiration dynamics. Figure 7e shows evolutions that are typical of the Mediterranean climate: the median values are higher than zero in all the months except the Summer ones with the maximum values attained again in November and December driven by the large precipitation amounts and the limited evapotranspiration demand.

**Figure 8:**
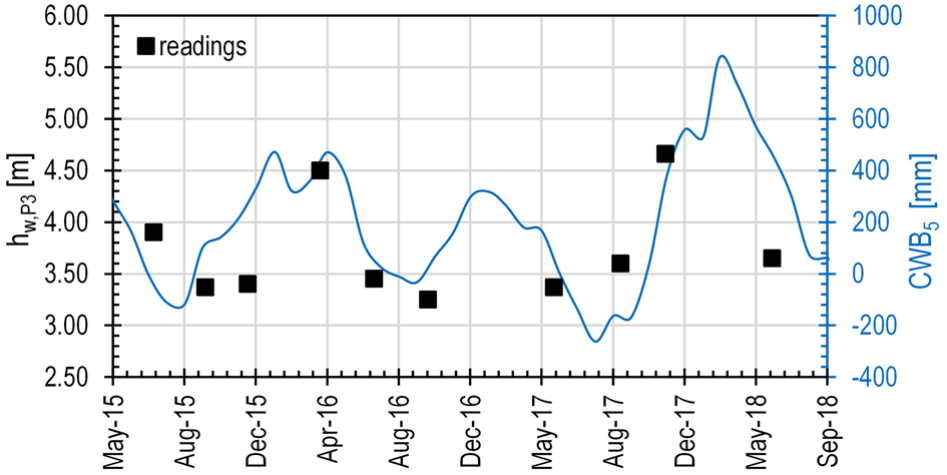
Pressure head, *h*_*w,P3*_, measured by piezometer P3 from July 2015 to May 2018, compared to the Cumulative Water Balance calculated over 5 months, *CWB*_*5*_ (modified after Manna^18^).

If *CWB*_*1*_ may return insights about the seasonal fluctuations in soil water budget, slope hydrology (e.g. soil water movement, water fluxes at the slope surface) is strictly related to the hydraulic properties of the soils in situ. In this regard, it is possible to retrieve how the fluctuating water levels evidenced a seasonal weatherinduced behaviour. On this topic, Rianna et al.^31^, after the analysis of the piezometric regime monitored within a similar slope in the Apennines chain, suggest to correlate the monitored oscillations to a soil hydrological balance of the area expressed through the *CWB* values over some months. In our case, Fig. 5 highlights a quite good correspondence between the trends of the readings (Fig. 5b–e) and those of the Cumulative Water Balance over 5 months, *CWB*_*5*_ (Fig. 5f–i). The delayed effect of some months on the pore-water pressure is, of course, a consequence of the overall low soils hydraulic conductivity. Particularly regarding the piezometer P3, featured by the longest running activity period, the fluctuation of the measured pressure head, *h*_*w,P3*_ (Fig. 8), is relatively well correlated to the *CWB*_*5*_ oscillations through the following equation

3 $$h_{w,P3} \;[{\text{m}}] = 3.5 + 0.0025 \cdot CWB_{5} \;[{\text{mm}}].$$

**Figure 9:**
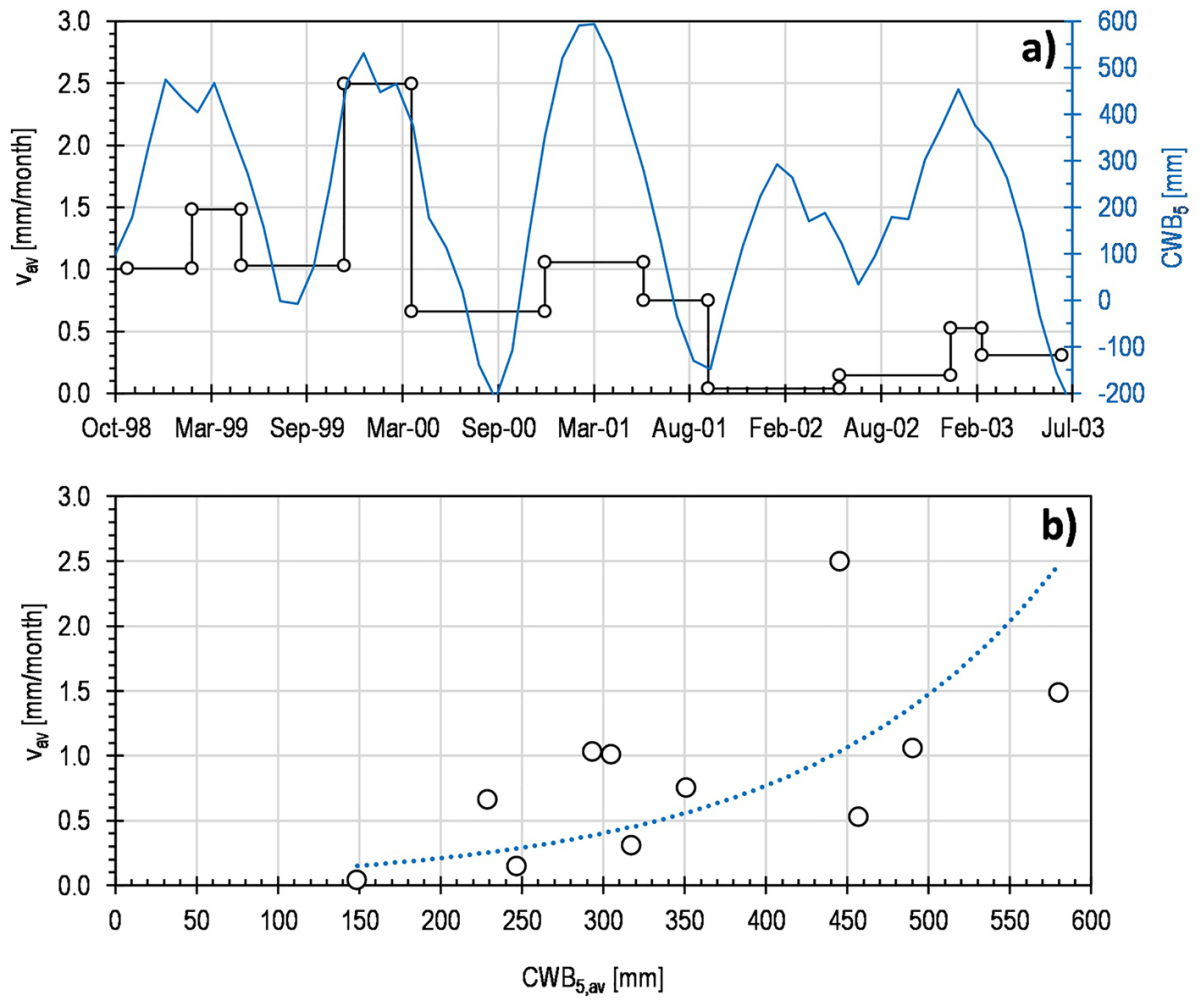
Displacement regime: (**a**) mean displacement velocity, *v*_*av*_, measured at the ground surface by inclinometer G1 from October 1998 to July 2003, compared to the fluctuating Cumulative Water Balance calculated over 5 months, *CWB*_*5*_; (**b**) *v*_*av*_ values compared to the Cumulative Water Balance over 5 months values averaged over the corresponding monitored period, *CWB*_*5*,av_.

On the other side, the same approach can be also reiterated to assess the potential role of weather forcing (by using *CWB* as proxy) on displacement regime. Particularly concerning the inclinometer G1, its highest continuity of operation allows us to have more reliable information about velocities at the monthly scale. As shown by Fig. 9a, they fluctuate between 0.1 and 2.5 mm/month, with acceleration/deceleration occurring during the wet/dry seasons. The detected variations seem again consistent with the *CWB*_*5*_ oscillations (Fig. 9a,b), thus revealing a delayed influence of the weather conditions on the kinematic response, as already observed on the piezometric regime. Figure 9a mostly shows how the negative *CWB*_*5*_ trend likely induced a movement slowdown in the examined period.

## Analysis of the failure mechanism

**Figure 10:**
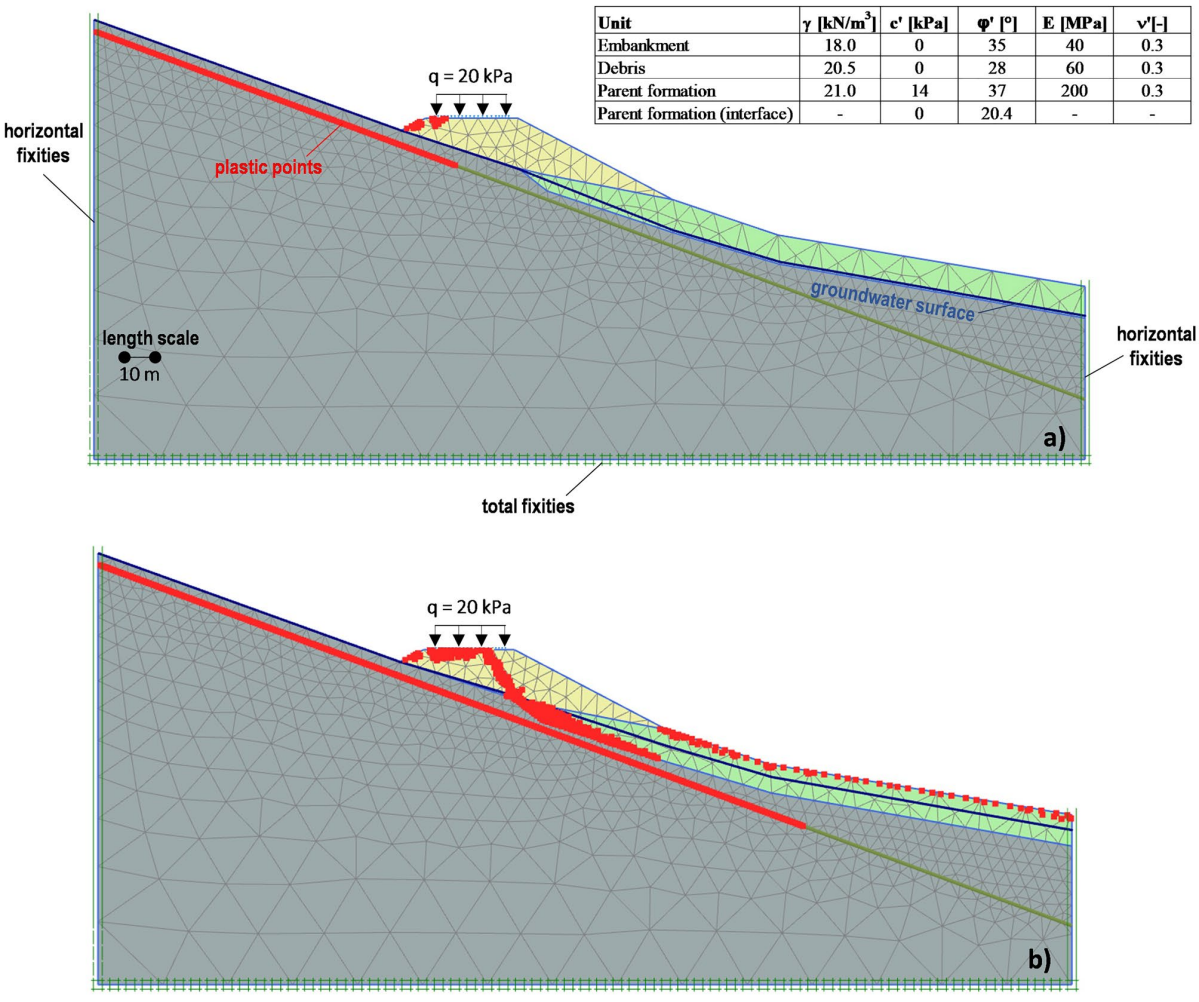
Results of the FEM numerical analyses (gravity loading): assigned soil properties; mobilized sliding surface assuming the minimum (**a**) and the maximum (**b**) detected groundwater level.

The monitoring data described in the previous section highlight that: (i) both the embankment and the foundation soils are affected by a failure mechanism; (ii) a weatherinduced seasonal pattern features their displacement rates. Such a mechanism has been checked by developing a simplified 2D numerical model using the finite element numerical code PLAXIS^32^. The model mesh consists of 1309 15-noded triangular elements (Fig. 10a,b). Furthermore, the soils are assumed to be homogeneous and isotropic, with an elastic-perfectly plastic behaviour whose properties are shown in Fig. 10a. Specifically, the mechanical properties of the embankment and parent formation have been derived from in situ and laboratory tests, while those assigned to the debris have been calibrated through the analyses. A continuous 20° inclined sliding surface within the parent formation has been simulated by interface elements featured by the residual friction angle measured in the laboratory.

**Figure 11:**
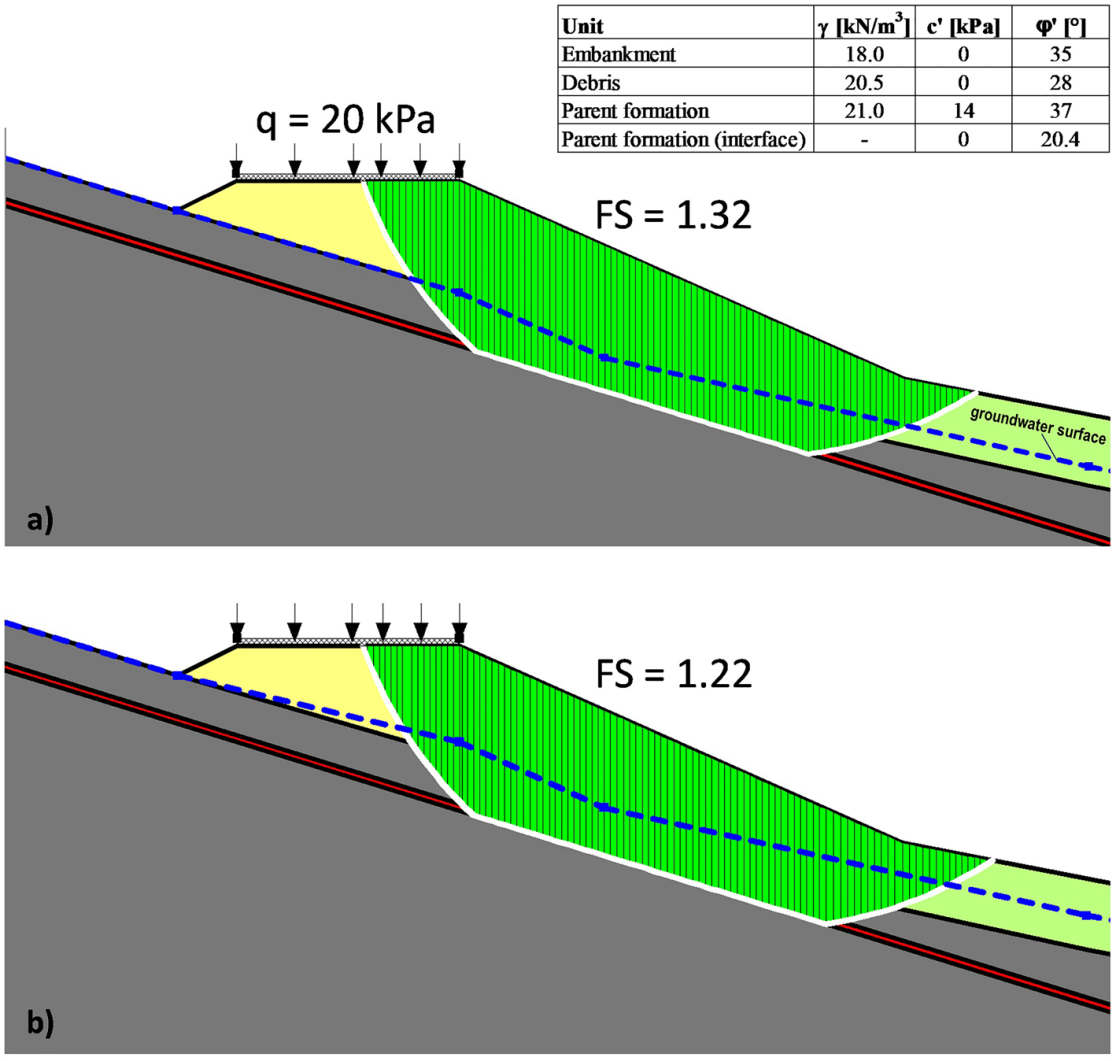
Results of the limit equilibrium stability analyses: calculated Factor of Safety, *FS*, assuming the minimum (**a**) and the maximum (**b**) detected groundwater level.

A first gravity loading analysis, consisting of applying the soil self-weight, has been performed. In addition, a constant vertical stress *q* = 20 kPa, uniformly distributed along the road width, has also been assigned to simulate the vehicle load. Regarding the groundwater surface position, both the minimum and the maximum detected water levels have been assumed (Fig. 5a). An overall equilibrium condition has been reached in both the examined cases. Such a result has also been confirmed by some 2D limit equilibrium stability analyses, based on the method of slices proposed by Janbu^33^ and carried out using the software SLOPE/W of the GeoStudio suite (Bentley Systems, Incorporated), that calculated a resulting safety factor *FS* ranging from 1.22 to 1.32 (Fig. 11a,b). Assuming nil the vehicle load (*q* = 0), the previously calculated minimum and maximum values have reached 1.24 and 1.34 respectively: the computed negligible *FS* increase has consequently highlighted the marginal role of the assigned surcharge.

The observed phenomena should therefore result from a local failure mechanism that, as shown by the number and positions of the activated failure plastic points calculated by the FEM analyses (Fig. 10a,b), is justified only assuming the highest groundwater surface position. At the same time, additional FEM analyses showed that the plastic points within the parent formation should not be activated if the peak strength parameters (c′ = 14 kPa; φ′ = 37°) were assigned to the interface elements, thus confirming that the corresponding sliding surface is very likely a pre-existing weak shear surface.

**Figure 12:**
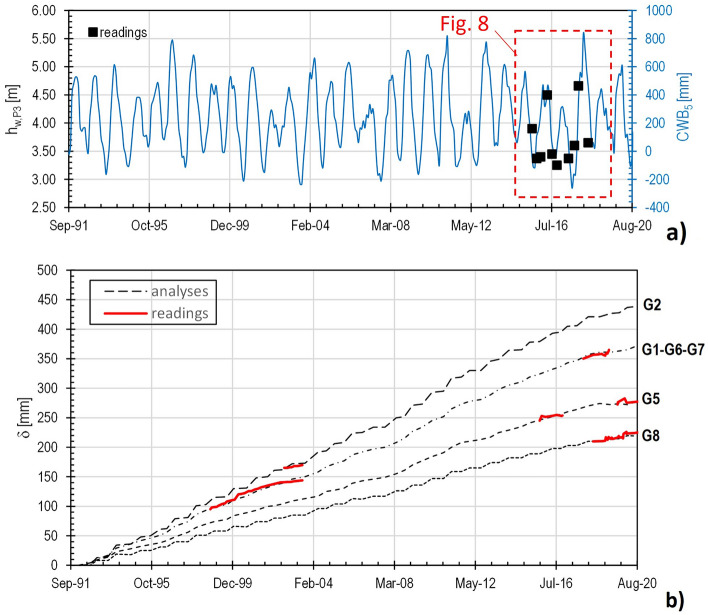
Results of the FEM numerical analysis aimed to find out the weatherinduced response from September, 1st, 1991 to August, 31st, 2020: (**a**) assumed pressure head fluctuation at piezometer P3, *h*_*w,P3*_, as a function of the Cumulative Water Balance over 5 months, *CWB*_*5*_; (**b**) cumulative horizontal displacements calculated on the ground surface at the four investigated vertical sections (G1–G6–G7, G2, G5, G8) compared to the corresponding readings (modified after Manna^18^).

**Figure 13:**
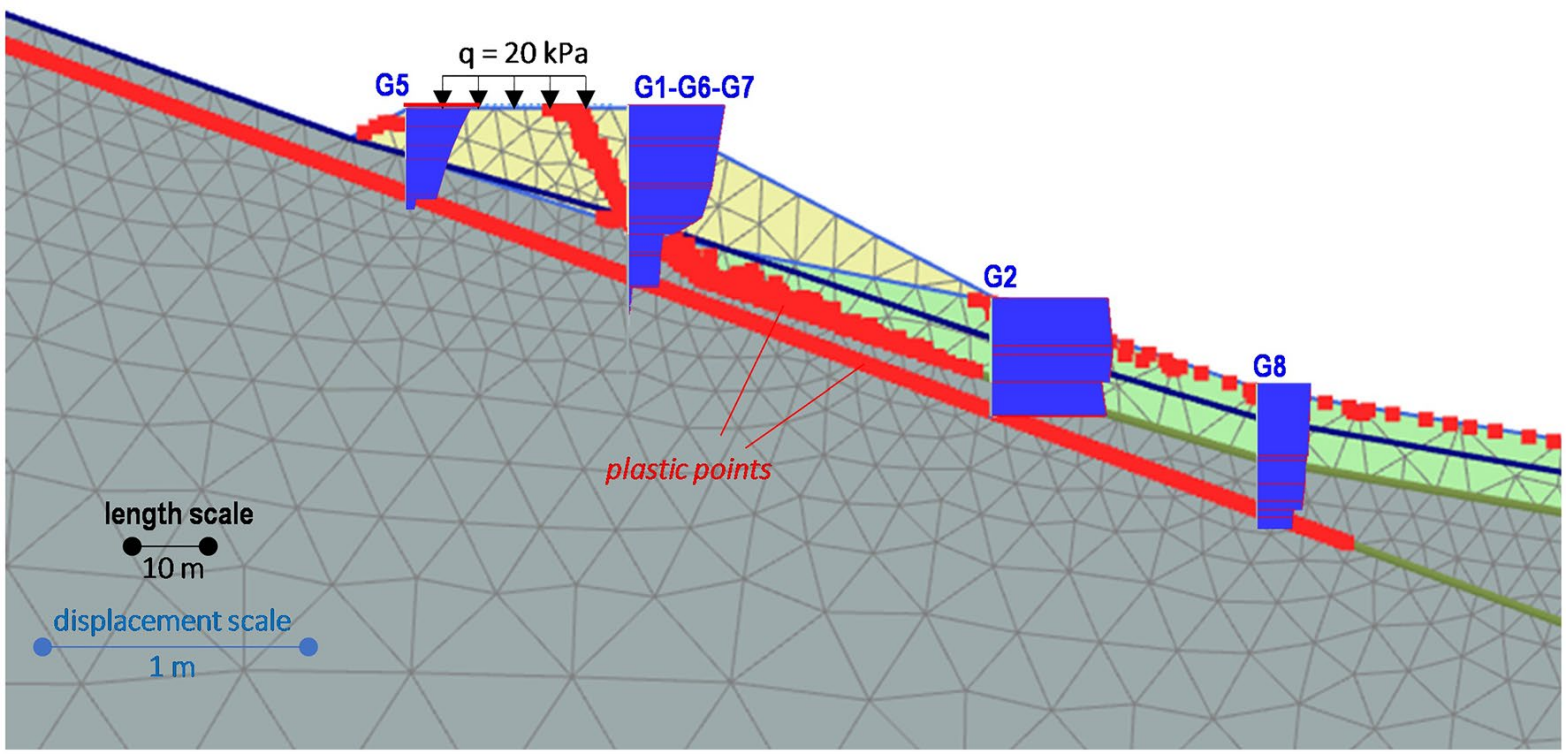
Results of the FEM numerical analysis aimed to find out the weatherinduced response from September, 1st, 1991 to August, 31st, 2020: cumulative horizontal displacements calculated at the end of the examined 30-year period at the four investigated vertical sections (G1–G6–G7, G2, G5, G8) (modified after Manna^18^).

Further FEM analyses have been launched to reproduce the weatherinduced slope kinematic response during the time period 1991–2020, more specifically from the beginning of the first considered hydrological year (September, 1st, 1991) to the end of the last investigated hydrological year (August, 31st, 2020). Such simulations have accounted for additional interface elements to allow relative displacements along the failure surfaces within the embankment and at the base of the debris revealed by the gravity loading analyses. These interface elements have been featured by the same properties of the corresponding crossed soils. Regarding the piezometric regime, to reproduce the changing with position of the groundwater table, it has been assumed the validity of the empirical Eq. (3) linking *CWB*_*5*_ to the pressure head *h*_*w,P3*_ tested from 2015 to 2018 for all the examined time interval (30 years). As a result of such an extrapolation, *h*_*w,P3*_ should have been affected by seasonal oscillations ranging from a minimum of about 3.0 m to a maximum of about 5.5 m (Fig. 12a). Before launching the numerical analyses, the following further assumptions, as derived from the monitoring, were also made: (i) the groundwater table follows the ground surface profile until the uppermost location of the embankment, and it is then linearly connected to the water level determined by P3; (ii) P3 and P4 are featured by the same pressure heads; (iii) the groundwater table is parallel to the top of the parent formation from P4 toward downslope. Figure 12b shows the cumulative horizontal displacements, δ, calculated on the ground surface at the four investigated vertical sections: they match with good approximation the corresponding values measured during the different monitoring periods. As shown in Fig. 13, the model also reproduces rather satisfactorily the shapes of the monitored inclinometric profiles, that are, of course, the combined effect of displacements concentrated along the failure surfaces and deformation occurring within the involved soils. In particular, it is interesting to observe the reproduction of the highest shear strain detected along both the slipping surfaces at the verticals G1–G6–G7 and G2 and along only the deepest failure surface at the verticals G5 and G8. Moreover, as shown by Figs. 12b and 13, δ increases in the slope direction from section G5 (δ = 28 cm) to G2 (δ = 44 cm), thus suggesting that the embankment might have experienced extension during his life. On the contrary, the following downslope reduction until the value calculated at section G8 (δ = 22 cm) suggests that the corresponding involved mass has been subjected to an overall compression state.

## Discussion and concluding remarks

The work has the main goal of proposing a framework permitting to assess long-term trends and the potential role of weather forcing on slope behaviour in areas where field monitoring is affected by significant gaps in temporal coverage. Monitoring activity could be indeed interrupted for not negligible time intervals, as in our examined case, by unexpected technical problems (e.g. failure of some installed devices) or by suspensions due to changes in local land management. To this aim, over the periods covered by monitoring, a simple relationship between variables (namely, piezometric and displacement regimes) and weather forcing, able to retrieve the main features of the seasonal variability, is calibrated. Cumulative Water Balance (*CWB*) over several months to account for specific soil hydraulic behaviour is exploited as proxy. After calibration, it can be used for blind-prediction over the periods not covered by monitoring data permitting to return information about the slope behaviour.

Particularly regarding the study case, the information collected from the described investigation and monitoring activities highlights that the slow movements affecting the analysed infrastructure results from a complex failure mechanism involving both the embankment and the sloping foundation soils. The results of some 2D FEM numerical analyses evidenced, in particular, that the deepest failure mechanism detected within the tectonised parent formation could be justified only assuming the mobilisation of a residual strength along a pre-existing surface. Given the weatherinduced seasonal variability of the detected movement, a simplified 2D FEM numerical model has also been developed to correlate the monitored kinematics to the piezometric regime estimated through a hydrological balance of the involved area. The latter has been, in turn, assessed according to the available weather data. The proposed model provided a continuous historical reconstruction of the kinematics, whose reliability (testified by the reproduction of the main observed mechanical features) suggests its use to evaluate the efficiency of some mitigation measures.

The proposed procedure is intentionally kept simple because it could serve as benchmark also in many other areas where the data of soil monitoring can be scarce and/or temporally inhomogeneous. In this perspective, another bottleneck (as in the investigated test case) could be the absence of monitoring data related to the weather variables (temperature and precipitation, at least); to deal with such constraints, we adopted in our case a national dataset (SCIA-ISPRA) while, at the same time, several international initiatives can provide the same information at global level with a proper spatial and temporal resolution. For instance, ERA5land, a downscaling from ERA5 reanalysis, provides hourly weather outputs and data related to the soil since 1950 up to the present time with a horizontal resolution of 9 km; 10.24381/cds.e2161bac. On the other hand, when reliable and long datasets provided by local weather stations are available in areas surrounding the investigated slopes, it could represent the best choice preventing all the inaccuracies and potential weaknesses associated with the adoption of gridded large scale datasets (e.g. they provide average information over the “cell” detected by horizontal resolution, for gridded observational datasets, thus it is often hard to understand which stations and in which periods inform the gridded dataseries).

Among the possible advances, *CWB* computation could be refined when key information about soil hydraulic properties and land cover are available. Indeed, in the present investigation, *CWB* adopts the algebraic sum between precipitation and potential evapotranspiration that are the two main forcings of the water entering/leaving the soil. However, the slope hydraulic behaviour could be strongly related to the “actual” water amount through the soil surface; it entails considering infiltration instead of precipitation and actual evapotranspiration instead of the potential one. However, if many expeditious predictive models are available in literature for assessing such the components, reliable evaluations need a proper knowledge of local features. Furthermore, the increasing availability of information about the displacements from remote sensing sources could support/complement the evaluation of long-term trends for test cases not sufficiently covered by in situ monitoring. Nevertheless, postprocessing and validation of the provided information require specific expertise and adequate computational resources.

## Data Availability

The datasets generated during and/or analysed during the current study are available from the corresponding author on reasonable request.
